# Epidermal Stem Cells in Hair Follicle Cycling and Skin Regeneration: A View From the Perspective of Inflammation

**DOI:** 10.3389/fcell.2020.581697

**Published:** 2020-11-09

**Authors:** Elena I. Morgun, Ekaterina A. Vorotelyak

**Affiliations:** Laboratory of Cell Biology, Koltzov Institute of Developmental Biology of Russian Academy of Sciences, Moscow, Russia

**Keywords:** hair follicle, stem cells, regeneration, wound, skin, inflammation

## Abstract

There are many studies devoted to the role of hair follicle stem cells in wound healing as well as in follicle self-restoration. At the same time, the influence of the inflammatory cells on the hair follicle cycling in both injured and intact skin is well established. Immune cells of all wound healing stages, including macrophages, γδT cells, and T_*regs*,_ may activate epidermal stem cells to provide re-epithelization and wound-induced hair follicle neogenesis. In addition to the ability of epidermal cells to maintain epidermal morphogenesis through differentiation program, they can undergo de-differentiation and acquire stem features under the influence of inflammatory milieu. Simultaneously, a stem cell compartment may undergo re-programming to adopt another fate. The proportion of skin resident immune cells and wound-attracted inflammatory cells (e.g., neutrophils and macrophages) in wound-induced hair follicle anagen and plucking-induced anagen is still under discussion to date. Experimental data suggesting the role of reactive oxygen species and prostaglandins, which are uncharacteristic of the intact skin, in the hair follicle cycling indicates the role of neutrophils in injury-induced conditions. In this review, we discuss some of the hair follicles stem cell activities, such as wound-induced hair follicle neogenesis, hair follicle cycling, and re-epithelization, through the prism of inflammation. The plasticity of epidermal stem cells under the influence of inflammatory microenvironment is considered. The relationship between inflammation, scarring, and follicle neogenesis as an indicator of complete wound healing is also highlighted. Taking into consideration the available data, we also conclude that there may exist a presumptive interlink between the stem cell activation, inflammation and the components of programmed cell death pathways.

## Introduction

As an outmost barrier of the organism, the skin has multiple functions, including a vitally important protective mission. Being exposed to an aggressive environment, it has developed a complex system of mutually dependent stem cell populations as well as immune response. Stem cells replenish regular tissue losses and are ready to upregulate their activity in case of injury. Immune system supports regenerative processes via direct interactions with invaders and clearance of wound debris. Also, it has the ability to intensify stem cell activity for production of differentiated progeny and restoration of lesions (Rahmani et al., 2020).

Epidermal homeostasis is maintained by stem cells that either self-renew or undergo differentiation forming the spinous, granular and cornified layers of the interfollicular epidermis (IFE) or multiple types of keratinocytes in the hair follicle (HF) generating the hair shaft (Gonzales and Fuchs, 2017).

The concept of epidermal stem cells (EpiSC) evolved from the assumption of equipotent basal cells of IFE toward the scheme of complicated hierarchy, kinetics, and behavior. Several approaches were proposed to determine stem cells in IFE and epithelial part of appendages. Among the most prominent characteristics is their ability to retain DNA label (for review see: Terskikh et al., 2012). Another widely accepted property of EpiSC is to form large clones (holoclones) *in vitro* in contrast to small abortive clones produced by their progeny as it was shown by pioneering experiments performed by Barrandon and Green (Barrandon and Green, 1987). Potten proposed the concept of proliferating epidermal unit containing slow-cycling stem cell in the center (Potten, 1974) for murine IFE where columnar structures could be observed at certain sites (Mackenzie, 1970). Labeling of EpiSC progeny in mouse epidermis confirmed the clonal organization of IFE (Ghazizadeh and Taichman, 2001). At the same time, the hypothesis of a single population of progenitor cells in homeostatic IFE of mouse tail with no role for stem cells was proposed (Clayton et al., 2007). The authors pointed out that the stem cell population undetectable in steady-state conditions might be recruited in wound healing. To identify interfollicular EpiSC, β1- and ɑ6-integrins as well as keratin 15 (Krt15), LRIG1, and MCSP were utilized as markers (Solanas and Benitah, 2013).

The most potent EpiSC reside in a specialized niche of HF named the bulge, where label-retaining cells were found by landmark paper of Cotsarelis with colleagues (Cotsarelis et al., 1990). They provide continuous cycling of HF and its regeneration (Jahoda et al., 1996) as well as the source of epithelial cells in the course of skin regeneration. The bulge zone of HF contains several subsets of EpiSC with diverse functions and regenerative potential. Pioneering experiments by Cotsarelis and his group revealed Krt15 as a putative marker of bulge cells (Lyle et al., 1998; Liu et al., 2003). *In vitro* lineage tracing showed that the progeny of Krt15 + cells contribute to all HF epithelial cell lines (Morris et al., 2004). Later on, the list of bulge markers was supplemented with CD34, Keratin 19, Lgr5, Gli1, Hopx, Lhx2, Nfatc1, Sox9, Tcf3/4, integrin α6, and Lhx2 (Rompolas and Greco, 2014; Gonzales and Fuchs, 2017). Another region of HF, the isthmus, contains cells with stem-like properties. They are expressing Lrig1, Gli1, MTS24, and Lgr6. Lrig1 + cells of the isthmus are involved in the infundibulum regeneration, at the top of which there is a population of cells expressing Sca-1 (Rompolas and Greco, 2014). The secondary germ is believed to be another source of HF renewal (Panteleyev et al., 2001).

Healthy human and mouse skin is populated by several types of immune cells such as dendritic cells, innate lymphoid cells (ILCs), T lymphocytes and macrophages (Mansfield and Naik, 2020), as well as mast cells and neutrophils (Nakamizo et al., 2020). Immune cells that are located above the basement membrane include CD8 + resident memory T cells (T_*RM*_), Langerhans cells, ILCs, and dendritic epidermal DT cells (DETCs). The dermal immune cells are represented by macrophages located near blood vessels, and T cells, which include near-follicular effector and regulatory CD4 + T cells (T_*regs*_), and γδT cells. Thus, each type of immune cell is situated in a specific compartment of the skin, and this is achieved by the homing to the corresponding microenvironment – different fractions of the HF and IFE keratinocytes have diverse chemokine signatures (Mansfield and Naik, 2020). It was shown *in vivo* and *in vitro* experiments that T_*regs*_, which express CCR6 on their surface, migrate to CCL20 ligand, derived from infundibulum cells (Scharschmidt et al., 2017). A similar mechanism of homing is implemented in the case of ILCs migration toward the upper part of HF. However, in addition to the CCR6-CCL20 interaction, ILCs are attracted to the epidermis by cytokines IL-7 and TSLP (Kobayashi et al., 2019). CD10-expressing DETCs, as well as T_*RM*_ are recruited to IFE cells due to the expression of CCL27 (Morales et al., 1999; Jin et al., 2010). Thus, the steady skin state is immunologically active, and there is an interplay between keratinocytes and inflammatory cells. Subsequently, EpiSC is in close interaction with cells of the immune system and are able to recruit them when the tissue is damaged (Naik et al., 2018).

Wound healing begins with an inflammatory phase involving cells of the immune system. Macrophages and neutrophils are the first and foremost, which secrete inflammatory mediators and phagocytize debris disinfecting the wound bed and enabling its further successful closure (Eming et al., 2007). Wound regeneration is incomplete without wound resurfacing, i.e., re-epithelization (Santoro and Gaudino, 2005). Dermal part of the skin is subjected to active regeneration and reorganization during wound healing and affects its outcome (Rippa et al., 2019). Blood and lymphatic vessels which supply the skin with nutrients and replenish immune cell pool in steady-state are extensively reorganized during wound healing providing proper regulation and structural reconstruction of damaged tissue. The effectiveness of wound healing correlates with the phase of HF cycle. It was found that skin containing anagen HF regenerates more effectively than that with HF in telogen. Wounds of mice anagen skin showed improved angiogenesis, increased proliferation of keratinocytes, accelerated transition to terminal differentiation, and ameliorated matrix synthesis, while telogen skin demonstrated an increase in the number of neutrophils and macrophages in the granulation tissue, as well as a high expression of macrophage migration inhibitory factor (Ansell et al., 2011).

During wound healing, epithelial HF stem cells (HFSCs) provide both HF neogenesis and wound re-epithelialization (Rompolas and Greco, 2014). HF regeneration in the middle of a full-thickness wound in mice is similar to embryonic hair morphogenesis. It is called wound-induced hair neogenesis (WIHN) (Wang et al., 2017; Gong et al., 2018). Formation of new HFs in adult skin may also be induced in experimental conditions by ectopic activation of specific signaling pathways (Sun et al., 2020). However, WIHN is appropriate only for mouse wound healing, while most mammals, including humans, do not regenerate skin appendages (Lim et al., 2018).

Activation of EpiSC is important condition for restoration of the structure and functions of the skin after injury. We will overview the characteristics of EpiSC, immune cells and inflammatory-related agents in the context of their interactions and functioning during wound healing and HF morphogenesis. As wound healing and inflammation are tightly connected with cell death, we tried to make brief references to possible interlinks with stem cell activation, where data are available.

## EpiSC Plasticity During Wound Healing Under the Influence of Immune Reaction

Since the skin delimits the body with the environment, it often undergoes damage and healing, and keratinocytes meet a first thrust (Proksch et al., 2008). The inflammatory milieu in the wound gets cells ready for re-epithelialization affecting their migratory and proliferation activities. Inflammation-induced cell dedifferentiation and can modulate cell stemness. However, the details of cellular behavior in the course of re-epithelization are not known at the moment. There is experimental evidence in favor of at least two models. According to the first, the cells of the basal layer migrate to the wound bed and then differentiate into suprabasal, and according to the second, suprabasal cells move from wound edges to the wound bed due to “leapfrog” mechanism and become basal (Rognoni and Watt, 2018). Thereby, epidermal cells possess certain plasticity: they can undergo de-differentiation as well as differentiation under the influence of accompanying wound healing inflammatory agents (Haensel et al., 2020). Noteworthy, similar transitions are known in inflammatory diseases. For example, exposure to IL-17 and IL-22, interleukins, which are elevated in psoriasis (Kim and Krueger, 2015), caused the expression of stem markers p63, CD44, CD29, and CD49f in keratinocytes *in vitro* due to activation of the RAC1/MEC/ERK/NF-κB pathway (Ekman et al., 2019). Nelson and colleagues found that activation of TLR3 by polyriboinosinic-polyribocytidylic acid (polyI: C), simulating the viral dsRNA in keratinocyte cell culture (Takada et al., 2017), causes a loss of Keratin1 and filaggrin (markers of differentiated keratinocytes) and an increase in the expression of Lgr5 and Lgr6 HF progenitor markers as well as Krt15 (Nelson et al., 2015). Besides, the participation of IL-6/STAT3 pathway in the acquisition of stem status is indirectly confirmed by the fact that STAT3 phosphorylation under the influence of IL-6 family members IL-11 and Oncostatin M (OSM) contributes to WIHN (Nelson et al., 2016b_B). There is experimental evidence that TA isoforms of stem/progenitor marker p63, which expression is affected by IL-6/STAT3, are involved both in the inhibition of keratinocytes ability to differentiate and in the stimulation of WIHN (Nelson et al., 2016a_A).

In addition to the acquisition of a stem marker profile by mature keratinocytes, other data are confirming the ability of epidermal cells to de-differentiate under the influence of the wound environment. Haensel and colleagues using RNA velocity analysis showed a significant proportion of *col17a1*^*Hi*^ basal cells and spinous cells of the SP1 population of wounded mice to demonstrate faster RNA dynamics than those from non-wounded skin. This indicated increased plasticity of cell fate in the skin sample of wounded mice with bidirectional transitions in several cell states between basal and spinous cells, which was not observed in the non-wounded animals. Thus, the wound inflammatory milieu stimulates epidermal cell plasticity and enfeebles restrictions on cell differentiation compared to their non-wounded counterparts (Haensel et al., 2020).

The implication of bulge HFSCs in re-epithelization has been established for a long time. However, different strategies for HFSCs labeling and a variety of markers employed led to a conclusion about different inputs of HFSCs subpopulations into the process (Garcin and Ansell, 2017). Some HF cells have been shown to play a dual role. In mice, Lgr6 + cells of the isthmus area provide ongoing skin restoration, including WIHN (Snippert et al., 2010). However, it seems now that both Lgr6 + and Lgr5 + stem cells are involved in IFE regeneration. Infundibulum Sca-1 + cells and Gli + stem cells are also involved (Brownell et al., 2011; Rompolas and Greco, 2014). HFSCs move downward for the regeneration and neogenesis of HF, while their upward migration contributes to re-epithelization. In spite of intuitive imagination of EpiSC as attached tightly to the niche, they probably may be highly motile making input into morphogenesis far from their original location. Nanba and co-authors showed *in vitro* that human EpiSC could be identified by their motility: the faster the cells move, the higher “stemness” they possess (Nanba et al., 2015). Another activity of HFSCs is linked to the barrier maintenance directly in the bulge niche where they are interacting with recruited immune cells to overcome telogen cell cycle inhibitory signals (Lay et al., 2018).

To accomplish regeneration, EpiSC can undergo reprogramming. Experimental data of Joost et al. suggest that inflammatory environment affects the expression of “follicular” (*Lgr5, Cd34, Cxcl14, and Sparc*) and “interfollicular” (*Krt14, Ifitm3, Eef1b2, Ly6e*) markers. Wound healing experiment with subsequent single-cell analysis showed that after wounding, the Lgr5 + progeny, while still in the bulge, begin to express both interfollicular and HF markers. However, interfollicular markers are upregulated; meanwhile, bulge markers are downregulated. Besides, Lgr6 + progeny in the intact skin expresses numerous receptors for interaction with wound ligands, and when the skin is damaged, their number slightly changes. Lgr5 + progeny expresses a few such receptors; however, when wounded, their number increases. This suggests that Lgr5 + progeny may become competent to interact with the wound environment at the level of Lgr6 + descendants. Moreover, this early upregulation of genes makes Lgr5 + progeny motile, as the number of genes (*Cd44, Cd9, Itgb1, Itga3*, and *Itga6*) that begin to be expressed are associated with cell migration (Joost et al., 2018), which indicates acquisition of the ability to re-epithelization. Thus, epidermal cells, beyond classical differentiation of basal cells to suprabasal ones, may change their status by loss of their maturity or reprogramming. from follicular to interfollicular state. Whether interfollicular cells can be reprogrammed into follicular cells under the influence of inflammation and what conditions determine the direction of differentiation/dedifferentiation/reprogramming, remains unknown and requires further research. Intriguingly, cells stemness was induced by inflammatory pathways, which intersected with programmed cell death pathways to some extent. It was mentioned above that de-differentiation of mature keratinocytes, and their acquisition of stem features may be associated with the influence of such an inflammatory agent as dsRNA, as well as upregulation of cytokines IL-17 and IL-22, which occurs during wound healing (Sasaki et al., 2011; Zhu et al., 2017). At the same time, the effect of dsRNA on TLR3 is a proven trigger of apoptosis and necroptosis (Seya et al., 2012) and upregulation of IL-17 and IL-22 in the psoriasis model is associated with a decrease in the expression of a RIPK-1, traditionally considered as a necroptosis component (Saito et al., 2018). Therefore, the stem status of epidermal cells may be affected not only by inflammation but also by the components of programmed cell death occurring during wound healing.

However, the relationship of epidermal cells with the immune system is two-component. On the one hand, inflammatory agents can affect epidermal cell activity; on the other hand, keratinocytes possess their own intrinsic immune activity. The basis of this phenomenon is the necessity to respond adequately to the inflammatory milieu that occurs as a result of injury or infection and to transmit a signal to surrounding cells. Epidermal cells are able to release IL-1, IL-6, IL -7, IL -8, IL -10, IL -12, IL -15, IL -18, IL -20, and tumor necrosis factor-alpha (TNF-α), which causes proliferation and differentiation of keratinocytes, production of cytokines by them, and migration of immune cells into the wound (Gröne, 2002). Innate immunity is a well-known characteristic of epidermal keratinocytes, presumably including those located in the basal layer. It was shown that interfolicular EpiSC could “remember” episodes of acute inflammation. Wound healing in mice previously exposed to imiquimod, which induced skin inflammation via TLR7-NALP3 axis, was more effective than in the control group. Moreover, this process was experimentally proven not to depend on macrophages or T cells. “Inflammatory memory” was based both on a transcription of the inflammasome pathway component *Aim2* in interfollicular EpiSC and the expression of its downstream effector *caspase-1* in post-inflamed skin. Caspase-1, in turn, is involved in the maturation and secretion of IL-18 and IL-1β, which were also increased in skin of the experimental animals (Naik et al., 2017). Therefore, previous “inflammation experience” could augment cutaneous wound healing due to EpiSC activity. Interestingly, all of the above agents are participants of the pyroptosis, the type of programmed cell death with morphological signs of necrosis (Vande Walle and Lamkanfi, 2016). However, Naik with colleagues did not show cell death in the experiment (Naik et al., 2017).

To summarize, immunocompetence of stem cells and changes in their stem status under the influence of inflammation are the two facets of the interaction of immunity and stem cells providing re-epithelialization after skin damage. As we can see, these processes involve various members of the TLR family. Activation of TLR7 is responsible for the “inflammatory memory” of interfollicular EpiSC, the influence of dsRNA on TLR3 causes the loss of differentiation markers by mature keratinocytes and the expression of stem cell markers Lgr5 and Lgr6 and Krt15. Therefore, immunoreactivity and the mechanism of dedifferentiation can also be interlinked. Furthermore, activation of pyroptosis and necroptosis also occurs through the TLR receptors. Consequently, various members of TLR family may be the “hubs” through which the pathways of immunity activation, stemness, and cell death can intersect.

**FIGURE 1:**
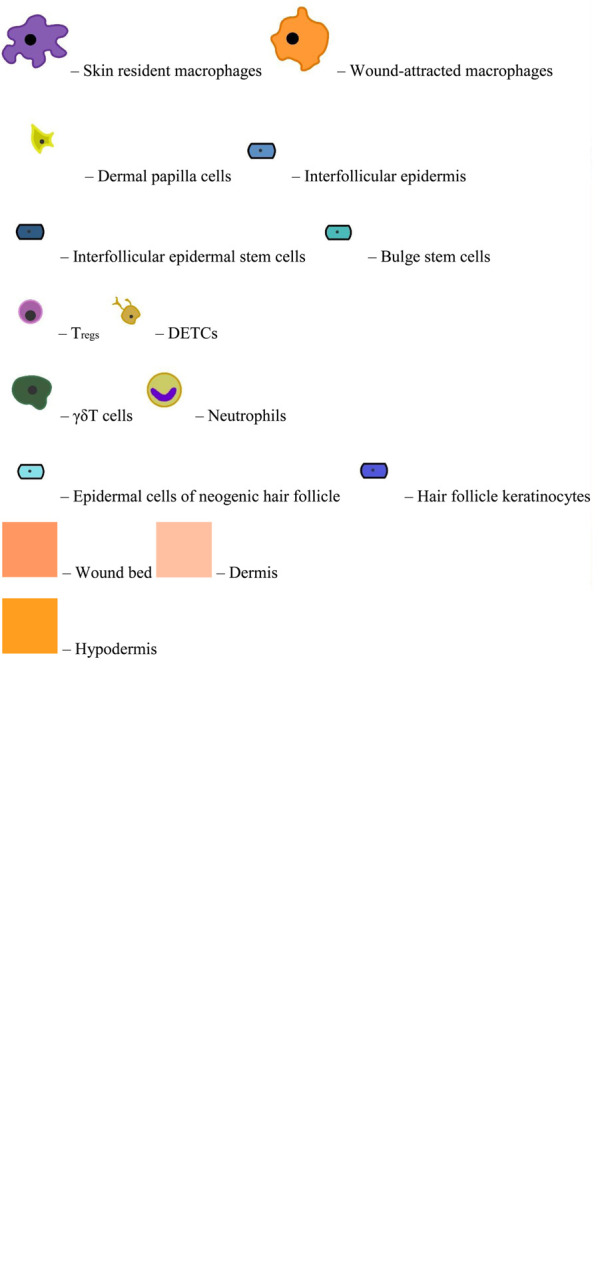
Facets of EpiSC plasticity under the influence of inflammatory agents.

Facets of EpiSC plastsicity under the influence of inflammatory agents is summarized in Figure 1.

## The HF Cycle and Immunity in Physiological and Injury-Induced Conditions

Cyclic changes in HF, namely, a continuity of successive phases of telogen, anagen, and catagen, partly occur owing to HFSC activity. Immune cell dynamics is associated with the transition of HF through the cycle and, in some degree, provides regulatory keys for it. We will discuss some striking features of this interconnection (for review see: Wang and Higgins, 2020) along with specific stage characteristics.

After the regression, HF passes into telogen, the phase of relative rest in terms of proliferation and biochemical activity (Paus and Cotsarelis, 1999; Krause and Foitzik, 2006). HF stays dormant until it is reactivated by intrafollicular and extrafollicular signals (Paus and Cotsarelis, 1999). Inactivity of HFSCs at this stage is provided mainly by bulge expression of BMP6 and FGF18 (Hsu et al., 2014). At the same time, telogen is supposed to be a stage of “progressive molecular activity”. In late telogen, the activity aimed at inhibiting quiescence predominates, whereas, in early and middle telogen, inhibition of cellular activity prevails. Also, there is a proliferative activity of several cell types: while bulge and secondary hair germ HFSCs are quiescent, keratinocytes from upper HF proliferate (Geyfman et al., 2015).

Anagen begins with the proliferation of Lgr5 + HFSCs, which are activated in late telogen. Hair germ HFSCs give rise to transit-amplifying cells that populate the germinative layer of the matrix. Outer root sheath (ORS) is formed by cells of the lower bulge (Welle and Wiener, 2016). It is well known that the key factors that induce anagen are the Wnt proteins, while the level of BMP signaling is downregulated by expression of its antagonist noggin (Botchkarev and Sharov, 2004). Sonic hedgehog, HGF, and FGF7, among others, support this process and stimulate the subsequent HF entry into anagen (Krause and Foitzik, 2006). In mice, anagen induction in response to exogenous stimuli and entry into the second anagen differ by underlying mechanisms, at least in terms of STAT3 signaling (Sano et al., 2000).

Upon completion of anagen, a controlled involution of HF occurs denoting onset of catagen, when most follicular keratinocytes undergo apoptosis (Paus and Cotsarelis, 1999; Krause and Foitzik, 2006; Welle and Wiener, 2016). At this stage, poorly dividing ORS cells create a new bulge, while slightly faster dividing cells form the secondary hair germ (Welle and Wiener, 2016). Unlike ORS and the hair matrix, the cells of DP do not die, though they express the suppressor of apoptosis bcl-2 (Krause and Foitzik, 2006). Toward the end of catagen, DP condenses and moves upward transiting into a resting stage to reside under the bulge (Paus and Cotsarelis, 1999).

Spontaneous HF cycle is mediated by different types of skin resident immune cells, which were listed above. Among multiple signals and niche components, stay of HF in telogen is regulated by FoxP3-expressing CD4 + T cells (also known as regulatory T cells, T_*regs*_) (Ali et al., 2017) and macrophages (Castellana et al., 2014). T_*regs*_ are associated with telogen. The abundance of these cells in telogen is threefold higher as compared to anagen. Moreover, their proliferative index correlates with the phase of the HF cycle: they are more active in telogen. Ali and colleagues found the bulge-associated subpopulation of T_*regs*_ (Ali et al., 2017). Disruption of adherence of the cell-cell junctions in bulge HFSC niche by E-cadherin removal activated proliferation program in telogen bulge HFSC repurposing new daughters to restore the breached integrity of the niche. In addition to cell cycle genes activation necessary during normal anagen, this proliferation activity needed elevation of the immune-signaling transcriptome. Notably, no transition to anagen was observed. Thus, proliferating stem cells were activated to restore the niche rather than to launch a new hair cycle. Proliferating HFSCs attracted immune cells, including T_*regs*_, increasing their numbers as compared to the resident skin population in a steady state.

Interestingly, this effect was independent of microbial presence. Moreover, immune cells, in turn, were necessary for activation of HFSC proliferation as the regular telogen proliferation-inhibitory cues were still active in the disrupted niche (Lay et al., 2018). Thus, telogen bulge cells are capable of proliferation independent of HF activation, and immune cells regulate this activity. The role of macrophages in telogen regulation is controversial. Not only they activate anagen but also prolong telogen: TREM2 + macrophages maintain the dormancy of HFSCs through OSM (Wang et al., 2019). Castellana and the colleagues showed that macrophage apoptosis promoted the entry of HFSCs into the anagen phase. As soon as the number of macrophages decreased due to liposome-induced apoptosis, HF of the experimental mice entered anagen, while the control group remained in telogen (Castellana et al., 2014).

As mentioned above, the telogen-to-anagen transition (TAT) is associated with macrophage apoptosis. Expression of *Wnt7b* and *Wnt10a* responsible for HFSC activation was elevated in skin resident macrophages during TAT. Moreover, experiments in the culture of mouse macrophages showed that their apoptosis occurs in parallel with the release of Wnts and that apoptotic macrophages stimulate the expression of Wnts by neighboring macrophages (Castellana et al., 2014). Interestingly, macrophages seem to produce extracellular vesicles containing Wnt3a and Wnt7b (Rajendran et al., 2020). Thus, the apoptotic reduction of macrophages during TAT is associated with the activation of the Wnt/β-catenin cascade (Castellana et al., 2014). Therefore, TAT and the Wnt/β-catenin cascade are related both to the activity of inflammatory cells and their apoptosis. Consequently, the activation mechanisms of HFSCs can be coupled with the pathways of programmed cell death.

Tissue-resident T_*regs*_ localized in close contact with HF sustain the bulge stem cells functions. Ali and colleagues demonstrated the role of T_*regs*_ in the activation of bulge HFSCs (Ali et al., 2017). In mice with T_*regs*_ depletion, a proliferative defect of the HFSC bulge zone during depilation-induced TAT was observed. In addition, lineage tracing showed a decrease in genes associated with the proliferation and differentiation of HFSCs (e.g*., Bgn, Bnc1, Cks2, Hmgn3, Gdf10*, and *Sox4*) in CD34 + integrin α6 (ITGA6)^*high*^ basal bulge HFSCs from depilated mice lacking T_*regs*_. When comparing the transcriptome of skin T_*regs*_ with those of skin draining lymph nodes (SDLN), it was shown that skin T_*regs*_ express Notch signaling ligand Jagged 1 (Jag1) 150 times higher than SDLN T_*regs*_ do (Ali et al., 2017). During anagen, the switching on Notch cascade activates Kit signaling and suppresses TGF-β establishing an optimal environment for the proliferation of matrix cells (Rishikaysh et al., 2014). Exogenous administration of Jag1 restores the proliferative function of stem cells in mice lacking T_*regs*_. Deletion of *Jag1* in T_*regs*_ reduces the expression of epidermal differentiation genes *Bgn*, *Ccnd1*, *Gdf10*, *Sox4*, *Sox7*, and *Timp3* as well as provokes anagen deterioration (Ali et al., 2017).

At the end of anagen, some significant changes occur in HF, in particular, downregulation of IGF1 (Krause and Foitzik, 2006). This growth factor is well known for its anagen-stimulating effect, as it decreases the expression of the negative regulator of anagen TGF-β1 and also promotes the proliferation of HF keratinocytes (Li et al., 2014). One of the sources of IGF1 is DETCs, the subpopulation of γδT cells that prevented epidermal apoptosis in a model *in vitro* (Sharp et al., 2005). Presumably, DETCs support anagen through secretion of IGF1 in three ways: through inhibition of TGF-β1, stimulation of keratinocytes proliferation, and downregulation of apoptosis, which is a key catagen pathway. Anagen ends with downregulation not only of IGF1 but also of other factors (including HGF, and FGF5) supporting anagen phase and with upregulation of hair growth inhibitors, such as TGF-β1, TGF-β2, FGF5 (Krause and Foitzik, 2006).

Catagen is induced by growth factors and cytokines, including FGF5 secreted by perifollicular macrophages and IFN-γ (Wang and Higgins, 2020). *Fgf5* mutations cause catagen retention in humans (Higgins et al., 2014). In certain pathological conditions, for example, in case of stress, mast cells were also shown to stimulate the onset of catagen in mice. In this situation, mast cell-deficient mice demonstrated catagen delay, as did knockout mice, which were not able to respond to the substance P produced by nerve fibers in stress conditions (Arck et al., 2005). Mature mast cells release a lot of significant inflammatory mediators and are involved in wound healing (Komi et al., 2020).

Interestingly, mouse vibrissal HF contains precursors capable of differentiation into competent mast cells *in vitro* and probably provide a niche for their differentiation *in vivo* (Kumamoto et al., 2003). Inflammation and HF cells in catagen are interconnected through cell death processes: apoptotic keratinocytes are cleared by macrophages in the mouse, whereas in humans, the macrophages themselves undergo apoptosis. In the experiment by Hardman with colleagues, a cyclic change in the number of perifollicular macrophages in a freshly isolated HF was shown with a minimal rate in late catagen (Hardman et al., 2019). The close interdependence between inflammatory cytokine TNF-α, Keratin 17 and apoptosis in keratinocytes was shown in the next study (Tong and Coulombe, 2006). In *Krt17* ^–/–^ mice, alopecia developed in the first week after birth due to increased apoptosis. *Tnf* ablation led to catagen delay. It is thought that apoptosis in *Krt17* ^–/–^ keratinocytes occurs due to accelerated sensitivity to TNF-α. In turn, apoptosis in *Krt17*-null keratinocytes led to premature onset of catagen. Consequently, cytoskeletal protein Keratin 17 is responsible for HF detention in anagen, meanwhile TNF-α leads to anagen-catagen transition. Tong and Coulombe suggest that Keratin 17 interacts with the TRADD adapter necessary to transmit a signal from TNFR1 (Tong and Coulombe, 2006).

Thus, HF stays in a specific phase is regulated by various types of skin resident inflammatory cells. Macrophages and T_*regs*_ play a significant role in telogen and anagen and by mast cells and macrophages in catagen. It is believed that during TAT, the induction of Wnt/β-catenin is associated with the apoptosis of macrophages, and activation of bulge HFSCs is associated with their induction by the T_*reg*_ cluster. However, it is possible that in certain conditions, inflammatory cells that are absent in normal skin contribute to the HF cycling.

In addition to the physiological conditions, TAT may be induced by injury. In the case of hair plucking, CCL2 is released, which leads to the recruitment of inflammatory macrophages and, as a result, the stimulation of anagen by TNF-α (Chen et al., 2015). Macrophages are also involved in another type of injury-induced TAT, which is called wound-induced hair anagen re-entry/growth (WIH-A), when, under the influence of a wound milieu, HF located at the edges of the wound switch to anagen (Abbasi and Biernaskie, 2019). It was shown that macrophage production of TNF-α invoked an activation of HF Lgr5 + stem cells being important for WIH-A. Suppression of these inflammatory cell infiltration resulted in the loss of WIH-A ability in HFs. To assess the role of macrophages in WIH-A, Wang, and co-authors eliminated these cells with clodronate liposomes (Wang et al., 2017). It led to an inability of the HF to proceed to WIH-A. Furthermore, an *in vivo* experiment performed to assess the role of TNF-α in these processes showed that *Tnf*^–/–^ mice did not develop HF anagen on post wound day (PWD) 15. In this study, *Lysm^*cre*^/:Tnf ^*flox/flox*^* mice with macrophages and neutrophils unable to express *Tnfa* were used. Skin regeneration in these mice occurred with a significantly reduced number of anagen HF, but an injection of lipopolysaccharide stimulated macrophages induced anagen. There is experimental evidence that activation of RAC-alpha serine/threonine-protein kinase (AKT) in Lgr5 + cells plays an essential role in the induction of TAT of HF. Knockdown of *Tnfr1* in Lgr5 + cells caused a decrease in TNF-induced p-AKT activity in cell culture. It is assumed that TNF-α activates β-catenin through the PI3K/AKT/mTOR cascade, and phosphorylation of β-catenin, in turn, is induced by AKT in Lgr5 + HFSC (Wang et al., 2017). There is also another way in which macrophages could cause anagen during skin damage. These cells secrete growth factors (Werner and Grose, 2003) that lead to activation of the PI3K-Akt-mTOR pathway involved in cell growth and survival (Fang et al., 2013) in epithelial cells during wound healing (Squarize et al., 2010). Activation of the mammalian target of rapamycin complex 1 (mTORC1) was observed in the bulge and hair germ cells in a growth phase-dependent manner suggesting its role in anagen activation (Kellenberger and Tauchi, 2013). Thus, macrophages are involved in both physiological TAT, plucking-induced anagen, and WIH-A in the described experiment; therefore, the contribution ratio of skin resident macrophages and those arrived at the wound bed is unknown (Rahmani et al., 2020).

However, there are indirect signs that, in addition to skin resident immune cells, wound cells that are completely unusual for intact skin can be involved in WIH-A. It can be supposed that the persistence of HFSC in anagen is regulated by polymorphonuclear leukocytes, as they can secrete reactive oxygen species (ROS) (Dunnill et al., 2017), and prostaglandins (PG) (Higgs et al., 1975), which are also involved in HF cycling (Valente Duarte de Sousa and Tosti, 2013; Zhao et al., 2015). PGE_2_, acting synergistically with PGF_2_, prolongs anagen and induces hair growth (Valente Duarte de Sousa and Tosti, 2013). PGE_2_ does not induce TAT but has a stimulating effect on early anagen HF manifested by increased hair length. PGF_2__*a*_ promotes protein kinase C (PKC) activation via phosphorylase C-associated receptors. Furthermore, PKC is upregulated in anagen, which indicates its significance for HF activity (Sasaki et al., 2005). ROS is an important mediator of inflammation being secreted during wound healing and contributing to wound disinfection (Dunnill et al., 2017). It was shown that adipose-derived stem cells are also able to secrete ROS this way promoting DP-induced hair growth and thus, may cause TAT (Choi et al., 2019). However, in an *in vivo* study during the transition from anagen to catagen, Zhao and colleagues showed elevation of ROS level by the Foxp1 protein via suppression of ROS scavenger Thioredoxin-1. This is also supported by the fact that administration of NAC antioxidant to *Foxp1* cKO mice led to the arrest of the HF cycle in anagen and increased HFSCs proliferation (Zhao et al., 2015). Thus, it is possible that in certain conditions, not only skin resident but wound-specific immune cells are also involved in the regulation of the HF cycle and morphogenesis. The relationship of the activation mechanisms of EpiSC with molecular cascades of wound healing will be considered in the next section.

## HF Neogenesis and Re-Epithelialization in the Course of Wound Healing

Widely recognized course of wound regeneration includes phases of inflammation, proliferation, and remodeling (Gurtner et al., 2008). Below we will consider these phases with critical elements of inflammatory reactions affecting epidermal cell activity, regeneration, and morphogenesis. Surprisingly, some of these elements demonstrate delayed effects in this respect. For example, macrophages acting in the inflammation phase (Eming et al., 2007) contribute to WIHN, which occurs during the time of the scar remodeling phase (Gay et al., 2020), and re-epithelialization begins in the proliferation phase under the influence of T_*regs*_.

### Inflammation Phase

*TNF alpha.* Platelets, leukocytes, macrophages, and mast cells are involved in the inflammation phase replacing each other. First of all, after a wound, platelets and polymorphonuclear leukocytes create a blood clot, and thus the tissue reaches hemostasis (Eming et al., 2007). Platelets form a clot interacting by GpIIb-IIIa receptors and adhering to the collagen of the disrupted subendothelium. Fibrin polymerization promotes the formation of a mature clot, which serves as a scaffold for infiltrating leukocytes and fibroblasts playing a sequential role in healing. Platelets also contribute to the subsequent stages of healing, for example, secreting catecholamines and serotonin, which act through receptors on the endothelium and cause vasoconstriction surrounding the wound (Gantwerker and Hom, 2011). In addition, platelets and polymorphonuclear leukocytes secrete TNF-α and IL-1 (Werner and Grose, 2003; Mussano et al., 2016) activating capillary endothelial cells and contributing to leukocyte transmigration (Bevilacqua et al., 1987). Neutrophils disinfect the wound eliminating debris and bacteria and provide antimicrobial protection by releasing H_2_O_2_, which produce ROS into the surrounding space.

Meanwhile, ROS not only protect the wound from infection, but they are also engaged in normal wound healing. ROS are involved in the proliferation of fibroblasts and vascular endothelial cells, promote proliferation and migration of keratinocytes, stimulate angiogenesis, and act as a chemoattractant for neutrophils, mononuclear phagocytes, eosinophils, basophils, and lymphocytes by stimulating the production of macrophage inflammatory protein (MIP)-1α (Dunnill et al., 2017). The importance of ROS for wound healing was confirmed in a study by Carrasco and colleagues, who showed the beneficial effects of ROS on wound healing and on HFSCs. The researchers induced transient ROS generation via the photodynamic effect as a result of the application of a photosensitizer (5-aminolevulinic acid (ALA) or its methyl derivative (mALA) cream) to mice in combination with red rays. This effect is used in clinical practice for induction of cell death in tumors. Generation of transient ROS in such a way led to the proliferation of bulge HFSC, as well as epidermal and dermal skin layers (Carrasco et al., 2015). ROS can also affect HF neogenesis through DP. Zheng et al. showed proliferation of DP cells and increased mRNA expression levels of vascular endothelial growth factor (VEGF)-A and glial cell line-derived neurotrophic factor (GDNF), leading to WIHN under ROS-induced hypoxia (Zheng et al., 2019). Also, one of the most critical cellular events mediated by ROS is mitochondrial apoptosis. When the mitochondrial membrane is permeabilized with ROS, apoptosis agents (cytochrome c, AIF, or Smac/Diablo) enter the cytosol (Circu and Aw, 2010), implementing relationship between HFSCs activity and wound healing through ROS as an inflammatory mediator and an apoptotic agent.

However, the role of neutrophils, ROS, and other inflammatory mediators produced by them is controversial. Dysregulation of neutrophils can lead to skin lesions. Abundant neutrophil infiltration into the wound bed is a key factor in the development of chronic inflammation, which underlies non-healing wounds. Excess of neutrophils causes overproduction of ROS, which provokes cell membrane and extracellular matrix (ECM) damage with subsequent premature cell aging (Zhao et al., 2016).

Neutrophils are also capable of recruiting macrophages leading to a transition from the neutrophilic stage of wound healing to the stage of macrophage infiltration (Eming et al., 2007).

Macrophages appear in the wounded skin after neutrophils and phagocytize them (Gurtner et al., 2008; Greenlee-Wacker, 2016). Macrophage infiltration is regulated by fibrin clot platelets, fibroblasts, leukocytes, a gradient of growth factors, pro-inflammatory cytokines and chemokines released by hyperproliferative keratinocytes along the margins of the wound (Werner and Grose, 2003). Macrophages express many receptors on their surface, for example, the TLRs of different types (Karin et al., 2006). In addition to phagocytic and antigen-presenting functions, they produce various growth factors, such as TGF-β, TGF-α, and VEGF, which regulate fibroplasia and angiogenesis (Werner and Grose, 2003). Macrophage ablation during the inflammatory phase in an experimental model of wound regeneration in mice was shown to delay wound healing.

In addition, macrophages are known to play an essential role in angiogenesis. There is a hypothesis, that they form “tunnels” in the ECM which are colonized by capillary sprouts, circulating progenitor endothelial cells or transdifferentiated endothelial cells during later stages of wound healing (Moldovan, 2002). Also, there are experimental data in favor of the fact that macrophages can also participate in lymphangiogenesis during wound healing: the number of macrophages reaches a peak during the period of migration and organization of lymphatic endothelial cells (Rutkowski et al., 2006).

It is well known that macrophages are polarized into two subtypes, M1 and M2, with different phenotypes (Xu et al., 2013) though continuum of intermediate states exists. M1 macrophages are CCR2 + cells (Fadini et al., 2013). They secrete pro-inflammatory cytokines and phagocyte dead cells and pathogens. They can acquire this phenotype under the influence of bacterial metabolites, IFN-γ, and a granulocyte-macrophage colony-stimulating factor (GM-CSF) (Xu et al., 2013). Anti-inflammatory M2 macrophages, which activation is regulated by IL-13 and/or IL-4 (Qing, 2017), secrete angiogenic factors and anti-inflammatory cytokines and stimulate wound closure (Xu et al., 2013). They express CX3CR1 (Fadini et al., 2013). In M2-deficient mice, a delay in healing is observed, which is most prominent in the late stages of regeneration (Minutti et al., 2017).

Macrophage ablation using diphtheria toxin (DT) leads to WIHN inhibition in mouse experiments. M2 macrophages produce follicle-stimulating growth factors, such as FGF2 and IGF1. These factors are thought to take part in WIHN, since they participate in the Wnt pathway (Kasuya et al., 2018). The study of Wang and colleagues, which was mentioned in the previous section, in contrast, showed the valuable role of M1 macrophages in WIHN. It was found that in Lgr5 + HFSCs, TNF-α released by M1 macrophages induced not only TAT and WIH-A but also WIHN through activation of AKT/β-catenin (Wang et al., 2017).

TNF-α is a critical player in the inflammatory response (Efimov et al., 2009). It is upregulated during the inflammatory phase of wound healing (Barrientos et al., 2008). In response to skin damage, TNF-α is secreted by polymorphonuclear leukocytes, then by macrophages over a period from 12 to 24 hours after skin injury (Xu et al., 2013). In keratinocyte culture, TNF-α and IFN-γ stimulate the secretion of growth factors (e.g., TGF- α), HLA-DR, interleukins (IL-1, IL-6 and IL-8), ICAM-1, and monocyte chemotactic and activating factor (MCAF) (Barker et al., 1991). During regeneration, TNF-α is expressed in proliferating keratinocytes along the wound margins (Xu et al., 2013). Exposure of a culture of keratinocytes to TNF-α led to the expression of chemokines, cytokines, growth factors, and cell surface receptors, which can *in vivo* attract hematopoietic cells, monocytes, macrophages, and neutrophils, as well as memory T-cells that provide an innate immune response (Banno et al., 2004).

Íîwever, the upregulation of TNF-α is a noticeable factor in the transition of the acute wound to non-healing status, including diabetic wound development (Siqueira et al., 2010; Xu et al., 2013; Zhang et al., 2017). In *in vivo* and *in vitro* experiments, TNF-α exhibits a pro-inflammatory and pro-apoptotic effect, as well as prevents scar formation, fibroblast migration, re-epithelialization and, as a result, prolongs the period of wound closure.

Overexpression of TNF-α leads to an increase in the number of neutrophils and macrophages in the wound bed through NF-κB signaling. Anti-TNF-α therapy helps to reduce the activity of NF-κB, to normalize the number of inflammatory cells, and to decrease the size of the wound bed. Experiments with mice knockout for the gene of TNF-α inhibitor, a secretory leukocyte protease inhibitor (SLPI), showed that upregulation of TNF-α induces matrix metalloprotease 9 (MMP9), which leads to collagen destruction and, as a result, abnormal healing (Ashcroft et al., 2012). In an *in vivo* experiment, application of recombinant TNF-α to a wound reduced the expression of genes associated with collagen synthesis (Salomon et al., 1991), while TNF-α induced the expression of collagenase in dermal fibroblast culture in several studies (Dayer et al., 1985; Brenner et al., 1989). In human keratinocyte culture, TNF-α induces the expression of gelatinase capable of degrading type IV collagen (Banno et al., 2004). Secretion of TNF-α in high doses for a long period harms healing because it suppresses the expression of TIMPs and ECM proteins and induces the expression of MMP-1, -2, -3, -9, -13 and MT1-MMP, thus leading to the degradation of ECM, the decline of cell migration, and collagen deposition. TNF-α contributes to non-healing wounds by increasing gelatinases, collagenases, and stromelysins levels (Barrientos et al., 2008). The result of therapy aimed at inhibiting TNF- α is not only a decrease in the inflammatory response but also the normalization of ECM synthesis (Ashcroft et al., 2012).

There is some evidence that TNF-α inhibits normal scar formation not only due to enzyme activity. It was shown in an *in vitro* contacting wound model that TNF-α suppresses TGF-β-induced expression of myofibroblast genes, such as α*-SMA, fibronectin type 1A*, and *collagen*. The authors confirmed that the effect of TNF-α is in the inhibition of TGF-β1-induced differentiation of myofibroblasts, which leads to the formation of a more fragile matrix with reduced contractile properties. It is thought that the high level of TNF-α in a non-healing wound prevents the differentiation of myofibroblasts and changes in the cytoskeleton configuration, which leads to the impairment in normal matrix contraction during wound healing (Goldberg et al., 2007). TNF-α also suppresses re-epithelialization during wound healing (Barrientos et al., 2008). The *in vitro* study showed that administration of TNF-α led to an elevation of the mRNA expression of IL-8, ICAM-1, and MCP-1 in HaCaT through PKCζ; however, TNF-α-induced decline in the proliferative and migratory activity of HaCaT cells did not depend on PKCζ (Zhang et al., 2017). In another study, exposure of human keratinocyte culture to TNF-α stimulated the expression of pro-apoptotic genes *BIK*, *BID*, and *TNFSF10* (Banno et al., 2004).

As one can see, TNF-α is not just an activator of the pro-survival and inflammatory pathways as well as the trigger of apoptosis and necroptosis, itis also involved in wound healing, stem cell activation, and in the HF cycling. Multiple complementary data are indicating that delicate balance of TNF-α expression and related signaling is required for proper skin restoration, including WIHN.

In addition to TNF-α, important mediators of inflammation during wound healing are PG secreted by damaged tissues under the influence of leukocytes and other immune cells (Higgs et al., 1975). The role of PG in wound healing is controversial. They activate apoptotic cascades and thus prevent excessive scarring because impaired apoptosis does not give fibroblasts and myofibroblasts disappear during the later stages of skin regeneration (Su et al., 2010). Dysregulation of PG levels causes both WIHN and wound healing abnormalities. Thus, in a model of a non-healing diabetic wound in *ob/ob* mice, a decrease in PGE_2_/PGD_2_ levels was shown, and leptin injections retained normal wound healing and proper levels of PGE_2_/PGD_2_ (Kampfer et al., 2005). However, not only their abnormal decrease but also increase can lead to aberrations in wound healing and HFSCs activation. It was shown that the expression levels of the prostaglandin D_2_ synthase enzyme (PTGDS) and its product PGD_2_ correlated with WIHN: high levels of PTGDS and PGD_2_ were interlinked with impaired neogenesis of HFs (Nelson et al., 2013). Despite the harmful effect of PGD_2_ on WIHN, there is a suggestion that another type of prostaglandin, PGE_2_, may induce WIHN through Wnt activation, as it was demonstrated in hematopoietic stem cells (Goessling et al., 2009; Gong et al., 2018).

Besides neutrophils and macrophages mast cells also take part in an inflammatory phase. They are a source of pro-inflammatory mediators and cytokines and modulate the number of neutrophils in the wound bed. The number of mast cells in the wound bed returns to its standard value within the first 48 hours after injury and then increases in the course of regeneration (Eming et al., 2007; Gurtner et al., 2008). As mentioned above, mast cells contribute to the regulation of the HF activity, namely, its entry into catagen (Arck et al., 2005).

To summarize, the inflammatory cells, which take part in wound healing, are mainly polymorphonuclear leukocytes (namely, neutrophil compartment) and macrophages. They and their mediators (TNF-α, ROS and PG) are involved not only in the regulation of the HF cycling but also in other activities of EpiSC including WIHN. Excessive inflammation alters HF homeostasis and wound healing process. It is expected that WIHN failure in this respect makes an additional indirect contribution to wound chronicity because of the importance of HF neogenesis in skin regeneration.

### Proliferation Phase

***T_*regs*_.*** This stage of wound healing is characterized by proliferation and migration of cells, mainly keratinocytes and fibroblasts (Gurtner et al., 2008). The transition from the phase of inflammation to the stages of proliferation and remodeling is controlled by immunomegulatory mechanisms. In particular, T_*regs*_ are accumulated at inflammatory sites and are involved in modulating the immune response. These cells secrete Amphiregulin, an epidermal growth factor-like growth factor that is associated with maintaining tissue homeostasis, inflammation, and immunity. Amphiregulin via PCLγ induces “inside-out” activation of the integrin-αν-containing complex, which causes the transition of TGF-β from an inactive form to an active one. Active TGF-β has a dual role: it suppresses excessive inflammation and makes mesenchymal vascular progenitor cells, pericytes, differentiate into myofibroblasts, thus contributing to angiogenesis (Zaiss et al., 2019). Also, during angiogenesis, endothelial progenitor cells arrive from the bone marrow to the healing tissue to form new vessels (Gurtner et al., 2008). Xu et al. (2013) and Qing (2017).

T_*regs*_ contribute to re-epithelialization, which occurs due to differentiation of HFSCs into IFE keratinocytes, their proliferation, migration to the center of the wound, and synthesis of a new basement membrane (Gurtner et al., 2008; Gantwerker and Hom, 2011; Mathur et al., 2019). Re-epithelialization is activated by growth factors of the HGF, FGF, and EGF families (Martin and Nunan, 2015).

Mathur and co-authors showed in *in vivo* experiments that T_*regs*_ stimulated differentiation of HFSCs into IFE through the control of the IL-17-CXCL5-neutrophil inflammatory cascade. In a model of subacute skin injury inflicted by tape stripping, T_*reg*_ depleted mice demonstrated thinner epidermis upon regeneration and downregulation of genes related to keratinocyte differentiation and stratum corneum formation. T_*regs*_ suppress the keratinocyte expression of *Cxcl5*, which is induced by the neutrophil regulator IL-17A. In the T_*reg*_-cell-lacking mice, neutralization of CXCL5 by antibodies restored differentiation, while IL-17A neutralization or co-depletion of neutrophils saved the migratory defect of Lgr5-derived cells in IFE during healing (Mathur et al., 2019). Thus, T_*regs*_ contribute to wound healing affecting a wide range of cells. In addition to initiating re-epithelialization, the fibrin matrix is replaced by granulation tissue consisting of fibroblasts, germinating vessels, and immature type III collagen in the proliferation phase (Gantwerker and Hom, 2011). Either T_*regs*_ can stimulate bulge cells and cause HF cycle changes in normal conditions or promote re-epithelization during wound healing due to immunomodulating properties.

Fibroblasts are the key players in this phase of wound healing. It is well-known that fibroblasts and myofibroblasts secrete procollagen molecules, as well as fibronectin, thus providing ECM development. Fibroblasts migrating from the edges of the wound under the influence of TGF-β, cell fibronectin, and endothelin-1 differentiate into myofibroblasts contributing to wound contraction (Xu et al., 2013). Nevertheless, there is another surprising way, in which fibroblasts contribute to wound healing: they can produce nitric oxide (NO), an inflammatory mediator, which is also involved in skin regeneration. Several facts are indicating that NO is associated with collagen production. First, *in vitro* wound fibroblasts produce NO, and inhibition of NO synthases (NOS) leads to a decrease in collagen production (Schaffer et al., 1997). Second, transfection of tissues of wounded rats with a plasmid containing murine inducible NOS (iNOS) increased collagen deposition (Thornton et al., 1998). Third, ablation of endothelial NOS (eNOS) in mice leads to prolonged wound healing (Lee et al., 1999). Besides, there is some evidence that NO can affect EpiSC activation. Zhu and colleagues treated burn wounds in rats with NO-gel with sodium nitrite and showed that this treatment improved both wound healing and follicular neogenesis. Application of the gel contributed to re-epithelialization of the wound, caused an increase in the number of procollagen-expressing fibroblasts by 40%. Also, blood vessels number augmentation and neutrophils and monocytes infiltration was observed in the wound bed. In the wound bed of the treated group, more HFs and CD34 + stem cells were observed (Zhu et al., 2008). Wolf et al. in an *in vitro* experiment showed basal production of NO by human DP cells. The authors suggest that NO secretion promotes hair growth by mediating the proliferation and differentiation of matrix keratinocytes surrounding the DP (Wolf et al., 2003).

However, we should note that excessive exposure of the wound to NO can have the opposite outcome. There are some data on the harmful effect of NO on wound healing, for example, the delay in wound healing under its influence in an aged rat model (Bauer et al., 1998).

### Scar Remodeling and Re-epithelialization Phase

During this stage, most endothelial cells, macrophages, and myofibroblasts undergo apoptosis or migrate from the wound bed, which consists of collagen and other ECM proteins. Type III collagen is replaced by type I collagen with the assistance of MMPs secreted by fibroblasts, macrophages, and endothelial cells (Gurtner et al., 2008). In this phase, HF neogenesis begins (Gay et al., 2020).

The mechanism of apoptosis that inflammatory cells undergo during healing remains under discussion. There is some evidence that the death receptor pathway mediates it due to TNFR activation, but some data indicate that the mitochondrial pathway via the anti-proliferative protein p53 is also possible. Fibroblasts die after they have synthesized a sufficient amount of fibers. And then, excessive endothelial cells are discarded (Greenhalgh, 1998). Thus, an involution of granulation tissue and retraction of blood vessels are observed (Gantwerker and Hom, 2011).

At the stage of remodeling, T cells are acting (Eming et al., 2007). Lee et al. showed that γδT cells are activated by IL-1α and IL-7, which are secreted by keratinocytes after injury (Lee P. et al., 2017_A). DETCs play diverse roles in wound healing. They contribute to the proliferation of keratinocytes by secretion of IGF1. In the mouse wound healing model, IGF1 secreted by DETCs caused a proliferation of keratin 14 + mouse epidermal cells and inhibited their terminal differentiation. IL-17A secreted by another subtype of γδT cells, Vγ4 T cells, can promote proliferation and terminal differentiation of mouse keratin 14 + epidermal cells (Zhu et al., 2019). There is also evidence of a stimulatory effect of KGF secreted by activated DETCs on keratinocyte proliferation. In addition to participating in the survival and proliferation of keratinocytes, activated DETCs affect inflammatory cells. They produce CCL3 and CCL4, which regulate the recruitment of inflammatory cells. DETC-derived KGF stimulates hyaluronan deposition by keratinocytes attracting macrophages (Ramirez et al., 2015).

γδT cells are the source of FGF9, which is involved in neofolliculogenesis in wounds (Gay et al., 2013). Gay with colleagues showed that *Fgf9* was upregulated in the dermis, just before the development of the hair placode during wound healing begins. The administration of antibodies against FGF9 into wound dermis decreased the number of newly formed HF. The number of γδT cells in the wound increased just before the raise of FGF9. The researchers showed that on PWD 12 Vγ4 + γδT population was the predominant source of FGF9, while DETCs was inactive in this respect. The study on *Tcrd*^–/–^ mice lacking γδT cells showed a significant decrease in the number of newly formed HF in the wound bed compared with control. An analysis of the timeline of *Fgf9* expression and *in situ* hybridization of wound skin at different time points allowed the authors to conclude that γδT cells are the primary source of FGF9 at PWD 10–12, which caused the expression of Wnt2a in dermal fibroblasts and thus led to the activation of Wnt pathway as well as to the expression of FGF9 in fibroblasts later on, and, consequently, increased Wnt activation in the dermis. Researchers found a small number of resident γδT cells in the human dermis with low density per area compared with the mouse dermis. Also, there were differences in the localization of γδT cells. In mice, cells were scattered throughout the dermis, usually at a distance from αβT cells and blood vessels. Human cells formed clusters with αβ T cells in vascularized dermal “pockets,” indicating that they rarely migrate between skin and blood. A small number of γδT cells in the human skin, as compared to mice, may explain the lower regeneration ability of human skin when injured (Gay et al., 2013).

The Wnt pathway makes an important contribution to *de novo* HF formation (Gong et al., 2018). Myung and co-authors used K14-CreER;*Wls*^*fl/fl*^ (WlsK14cKO) mice with knockout of *Wintless*, the gene, corresponding to Wnt secretion in the basal cells of IFE and HF. In the mouse model of wound healing, the absence of DP formation in the dermis of mutant animals was shown using alkaline phosphatase (AP) whole-mount staining (Myung et al., 2013). Transfection of human HFDP cells by negative Wnt regulator *CXXC5* led to downregulation of β-catenin, AP and PCNA, and knockdown of CXXC5 had the opposite effect. Lee and colleagues assume that Wnt3a activates CXXC5. Exposure of human DP cells to Wnt3a increased CXXC5 via a negative feedback mechanism. It is suggested that interruption of Wnt inhibitory signaling may stimulate WIHN, especially in humans (Lee S.H. et al., 2017_B). As it was mentioned above, in addition to γδT cells, the Wnt pathway can be activated by macrophages.

Lee and colleagues showed that γδT cells affect HFSCs not only indirectly through Wnt-mediated contributions to WIHN. These cells are an excellent example of the direct relationship between wound healing and HFSCs activation, as they stimulate the proliferation of bulge CD34 + HFSCs. The authors used the conditional knockout model for the *caspase-8* gene in epidermal cells for simulating wound healing to investigate the role of IL-1 in EpiSC activation during this process (Lee P. et al., 2017_A). Previously it was shown that *caspase-8* loss led to upregulation of IL-1α secretion because of an influence on caspase-1, which take part in the conversion of IL-1 precursor into an active form (Lee et al., 2009). Caspase-8 is widely known for its participation in the process of death receptor apoptosis, but besides, it has other functions. For example, it is involved in the processing of IL-1β, which contributes to inflammation (Gurung and Kanneganti, 2015).

Furthermore, IL-1β can indirectly influence HF: transplantation of IL-1β-stimulated bone marrow-derived macrophages into mice induced hair growth (Osaka et al., 2007). Lee and colleagues proposed the following HFSCs activation scheme: after wounding, the expression of caspase-8 decreases and, as a result, IL-1α is released from keratinocytes, which, together with IL-7, increases the population of γδT-cells (Lee P. et al., 2017_A). The latter stimulates the proliferation of bulge stem cells and mobilize them for wound healing. In inflammatory conditions keratinocytes also secrete CCL2 (Behfar et al., 2018), which attracts macrophages secreting IL-1β inducing hair growth. This mechanism potentiates the effect of IL-1 and IL-7/γδT cells on HFSCs proliferation (Lee P. et al., 2017_A). The above data once again proves the complicated interconnections between HFSCs activation pathways, inflammation participants, and cell death.

The scarring, which accomplishes the regeneration, much depends on the inflammatory process features, as well as on the expression of the cytokine profile (Liechty et al., 2000). However, scar development precludes restoration of the structure and functions of the wounded skin amply, not to mention severe complications such as keloids (Lim et al., 2018). Therefore, studies focusing on the possibility of regulating scar development via modulating the inflammation are of great practical importance. For example, in mice lacking T-lymphocytes, wounds heal without scars; however, in mice that do not develop both T- and B-lymphocytes, skin regeneration ends with scarring (Gawronska-Kozak et al., 2006). In the fetus, the skin regenerates without scars, since fetal wound healing occurs with a reduced inflammatory reaction, in part because macrophages, neutrophils, and mast cells of the fetus differ in size and maturity from those in adult tissues (Takeo et al., 2015). Martin et al. (2003) argue that inflammation is not prerequisite for effective wound healing if antibiotics stop the development of a bacterial infection. In the *pu.1 ^–/–^* mouse model, which does not develop functioning neutrophils and macrophages, skin regeneration is similar to fetal healing without the development of an inflammatory response and, as a result, under conditions of lower levels of TGF-β and IL-6. Thus, at the moment, information about the possibility of a direct influence on the scar through inflammation modulation is contradictory.

Nevertheless, studies are pointing out the possibility of indirect eliminating the effects of excess inflammation manifested by fibrotic scarring via agents produced by dermal cells. Lim and colleagues showed in their work that activation of Sonic hedgehog pathway leads to the formation of DP, which is a necessary condition for follicular neogenesis, which, in turn, leads to the restoration of skin functions at the site of injury and prevents the formation of scars (Lim et al., 2018).

Consequently, inflammation, scarring, and WIHN are interlinked. This is confirmed by fibrotic healing in WIHN^–^ mice wounds. In this type of wounds, unlike WIHN^+^ ones, late macrophages phagocytized a Wnt inhibitor SFRP4. In the late stages of wound healing, Wnt ligands paradoxically induced fibrotic healing, and these ligands are decreased in WIHN^+^ wounds. A similar process occurs in humans (Gay et al., 2020), where wounds lack WIHN and commonly heal with a cosmetic defect. Thereby, the role of Wnt ligands in wound healing is controversial: on the one hand, they can promote WIHN, which prevents scar formation and causes skin restoration; on the other hand, chronic expression of such ligands can cause wound fibrosis. Since macrophages phagocytize Wnt-inhibitor, it can be assumed that the chronic presence of Wnt ligands in the wound may be a consequence of prolonged inflammation, causing impaired regeneration and abnormal scarring.

At the moment, the involvement of different types of stem cells in HF neogenesis is under discussion (Plikus et al., 2015). There is evidence that first telogen Lgr6 + stem cells transplanted onto the backs of nude mice are capable of reconstructing HF (Snippert et al., 2010). Another study gives a key role in follicular neogenesis to bulge Lgr5 + stem cells. Depletion of Lgr5 + cells in Lgr5-Cre:R26^*DTR*^/ + mice resulted in WIH-A arrest on PWD 15 as well as a decrease in the number of neogenic HF by PWD 21-23 (Wang et al., 2017). However, another type of bulge stem cells, Krt15-expressing cells, are not involved in WIHN (Plikus et al., 2015).

Proper WIHN requires dermal papilla (DP) formation likely occurring by condensation of dermal fibroblasts or pre-existing progenitors. Experiments on *Blimp-1* knock-out mice demonstrated a violation of WIHN, suggesting an essential function of fibroblasts expressing Blimp-1 in DP neogenesis (Telerman et al., 2017). On the other hand, DP cells themselves take part in wound healing. Being one of the main components of the wound environment, TGF-β1 upregulates the expression of fibroblast-specific protein 1 (FSP1) and vimentin in DP cells and downregulated α-SMA expression suggesting that DP cells can differentiate into fibroblasts during wound healing (Bin et al., 2013). DP can also contribute to wound healing by secreting the anagen-inducing hormone leptin (Jimenez et al., 2015), which was shown to improve the migration and proliferation of keratinocytes as well as to accelerate angiogenesis (Tadokoro et al., 2015).

Thus, to sum up, inflammation during wound healing is like a double-edged sword. On the one hand, excessive inflammation leads to the establishment of long-term non-healing wounds. The optionality of inflammation for wound healing was shown by a series of experiments, for example, with the ablation of functioning neutrophils and macrophages. On the other hand, the release of the inflammatory cytokine TNF-α is a significant factor in WIHN, which is a prerequisite for scar formation without a cosmetic defect. Also, we can make one more conclusion about the potential intersection of wound healing and activation of HFSCs not only through wound healing cascades but also in other ways, for example, through programmed cell death. These considerations are prompted by data on the role of the upstream activator of apoptosis and necroptosis TNF-α and the downstream participant of apoptosis Caspase-8 (Varfolomeev and Vucic, 2018) in the activation of HFSCs as well as in normal and pathological wound healing.

## Discussion

Undoubtedly, there is a close interplay between the activation of EpiSC, specifically, HFSCs, as a whole and WIHN as its particular manifestation with the participation of wound healing via inflammatory cells, mediators, and dsRNA. Inflammation is involved in the regulation of HF cycling in intact skin and into the activation of HFSCs during wound healing. However, there is a reason to believe that the relationship between HFSCs functioning and wound healing has a much more complex regulation. We want to take a chance here and suppose that this regulation may comprise not only inflammatory agents but also components of the signaling pathways of cell death, including apoptosis and necroptosis. As it was discussed above, ROS, activators of pro-apoptotic and inflammatory cascades, together with the “external” apoptosis and necroptosis activators TNF-α and dsRNA, and the downstream mediator of apoptosis caspase-8 influence wound healing and HFSC activation. These findings are particularly interesting since apoptosis is classically considered to be a non-inflammatory way of cell death (Nagata, 2018). There are also new data suggesting that apoptotic cells are not inert: they may trigger genes associated with wound healing, proliferation, and a decrease in inflammation in adjacent cells (Medina et al., 2020). It is known that the signaling pathways of inflammation and cell death are interconnected through RIPK-1 and RIPK-3, including the situation of wound healing, as shown in several studies (Takahashi et al., 2014; Godwin et al., 2015; Moriwaki et al., 2015; Gupta et al., 2018). Such intersections can also occur in the HF. Moreover, it can be speculated that RIPK-1 and RIPK-3 play an unexpected role in HF dynamics. We found the cycle-related expression of RIPK-1 and RIPK-3 in mouse and human HF cells: It was found in the anagen HFs and absent in telogen ones (Morgun et al., 2020). As it was described above, hair growth may be promoted via dsRNA/TLR3/IL-6/STAT3 pathway (Nelson et al., 2015, 2016a_A; Nelson et al., 2016b_B). Based on Moriwaki data obtained in a mouse model of colitis, as well as in an experiment on bone-marrow-derived dendritic cells, it can be concluded that after LPS-induced stimulation of TLR4, RIPK-3 participates in the activation of the NF-κβ pathway, which leads to interleukin expression and STAT3 phosphorylation (Moriwaki et al., 2014). It may be speculated that RIPK-3-induced activation of NF-κβ is an additional intermediate link between the stimulation of the TLR3 and IL-6/STAT3- induced hair growth.

The regulation of inflammation and other processes that occur during wound healing is very complex and multidirectional. Excessive scarring can cause a cosmetic defect, while a delay in the formation of a scar (like in a non-healing wound) can cause severe complications during the wound healing process. The functions of HF in wound healing are also a two-way street: it is a source of stem cells both for re-epithelialization and for self-regeneration, while its neogenesis in the wound bed is essential for the functional recovery of the skin after injury. However, humans are unable to accomplish WIHN. Likewise, there is no adequate model for studying the damaged human HF. There are several *in vivo* and *in vitro* models admitting studies of human HF life activities. For example, researchers offer a humanized scalp model (Yamao et al., 2015), a SCID mouse xenotransplantation model (Oh et al., 2016), and *in vitro* culture model of a single human HF (Ma et al., 2016), and DP organoid model (Gupta et al., 2018). It is believed that the use of HF or their cellular components can significantly affect the course of wound healing, while *in situ* induction of hair growth during wound healing can stimulate epimorphic regeneration instead of scarring (Rippa et al., 2019). Attempts to transplant or “grow” HF in the wound bed have a long history. There is a classic work of the Jahoda with co-authors, where the induction of hair growth by transplantation of cultured DP cells in the lower part-amputated HF, was shown (Jahoda et al., 1984). Kageyama and colleagues showed HF development after the transplantation of bead-based HF germs, which were prepared with mouse embryonic mesenchymal cells or human DP cells and epithelial cells, into the back of nude mice (Kageyama et al., 2019). Lei and co-workers accomplish *in vivo* hair growth after transplantation of organoids, which were developed from mice neonatal epidermal and dermal cells (Lei et al., 2017). Lee and co-authors generated the artificial HF using mouse pluripotent stem cells in an *in vitro* study (Lee et al., 2018). Using skin epidermal keratinocytes and human DP cells we fabricated an artificial hair germ-like structures expressing a number markers, such as Lef1, EpCAM, and P cadherin attributed to folliculogenesis (Kalabusheva et al., 2017). Abaci and colleagues managed to compose a factitious human HF inside bioengineered skin constructs (HSCs) using 3D-printed molds. Transfection of DP cells with *Lef-1* restored the intact transcription signature of the DP and significantly increased expression of markers of the inner and outer root sheath, as well as the medulla, which indicated the efficiency of cell differentiation within the HSCs toward the HF. Moreover, human umbilical vein endothelial cells (HUVECs) were encapsulated into HSCs, and this resulted in HF vascularization and in the hair growth stimulation in immunodeficient mice (Abaci et al., 2018). Returning to the subject of our review, we suggest that engagement of inflammatory cells during HF-like structure fabrication may improve the outcome of transplantation. Notably, there could be used not only distinctive for skin in steady state immune cells, such as T_*regs*_ or γδT cells but also polymorphonuclear leukocytes (in particular, neutrophils) and macrophages. Thus, partial simulation of wound milieu may be helpful to reproduce WIH-A and WIHN microenvironment during hair transplantation into the scar, which is a promising method of its amelioration (Jung et al., 2013), or into the balding scalp. It may be proposed, that macrophages within the hair-bearing graft could improve hair growth by three ways: trough AKT/β-catenin activation (Wang et al., 2017); via induction of Wnt-signaling by dint of growth factors release (Kasuya et al., 2018), or production of Wnt-ligands upon their apoptosis activation (Castellana et al., 2014). Neutrophils, releasing ROS, could stimulate secretion of growth factors by DP cells and, thus, cause hair growth, maintain DP identity and support correct epithelial-mesenchymal interactions through the restoration of native DP signature (Zheng et al., 2019).

Thereby, the study of the components of inflammatory cascades as general targets for the treatment of wound healing and pathologies assumes a new significance. According to EpiSC plasticity, it may be proposed, that inflammation milieu can promote HF development from cultured cells transplanted into the wound. However, inflammation- induced effect on stemness can not only ameliorate wound healing but also cause diseases associated with pathological cell proliferation and differentiation failure, for example, psoriasis. Likewise, several germinative epidermal cells have an “inflammatory memory”, which in the experiment leads to better healing, though the long-term outcome of this approach in humans is unknown. In addition, it is known, that skin pathologies characterized by excessive inflammation such as epidermolysis bullosa (Mallipeddi, 2002) and burns (Koh et al., 2014) improve the risk of squamous cell carcinoma. It can be assumed that inflammation so radically affects the homeostasis of the epidermis that differentiated epidermal cells acquire stem features, and epidermal SC overreact, resulting in malignant skin degeneration. This is another indirect evidence of the ambiguity of the effect of inflammation on the differentiation status of epidermal cells. Consequently, using TNF-α, ROS, and NO, as well as cells, which release these inflammatory agents, can be problematic, as they can cause quite severe skin pathologies. Thus, it is necessary to find a delicate regulation of inflammatory cascades.

## Author Contributions

EM analyzed the literature and wrote the manuscript. EV edited the text and proposed ideas. Both authors contributed to the article and approved the submitted version.

## Conflict of Interest

The authors declare that the research was conducted in the absence of any commercial or financial relationships that could be construed as a potential conflict of interest.
